# Determinants of Oral Health‐Related Quality of Life Among Individuals Requiring Endodontic Treatment

**DOI:** 10.1155/ijod/2026348

**Published:** 2026-06-30

**Authors:** Jishnu Pradeep, Kalyana-Chakravarthy Pentapati, Shashi Rashmi Acharya, Ravikiran Ongole

**Affiliations:** ^1^ Department of Public Health Dentistry, Manipal College of Dental Sciences, Manipal Academy of Higher Education, Manipal, India, manipal.edu; ^2^ Department of Conservative Dentistry and Endodontics, Manipal College of Dental Sciences, Manipal Academy of Higher Education, Manipal, India, manipal.edu; ^3^ Department of Oral Medicine and Radiology, Manipal College of Dental Sciences Mangalore, Manipal Academy of Higher Education, Manipal, India, manipal.edu

**Keywords:** caries, oral health, quality of life, Sustainable Development Goal 3 (good health and well-being)

## Abstract

**Background:**

Dental pain, swelling, and sensitivity are typically assessed by dentists, who often overlook their impact on patients’ daily lives. “Oral health‐related quality of life” (OHRQoL) assesses the impact of oral conditions on daily activities. Previous studies have shown that dental issues have a significant effect on OHRQoL, motivating patients to seek treatment.

**Aim:**

To evaluate OHRQoL and its associated factors among patients requiring endodontic treatment in the Indian context.

**Methodology:**

This cross‐sectional study included patients aged 18 years or older who required endodontic treatment. We excluded patients with acute conditions or communication barriers. Sociodemographic data, clinical variables, self‐reported pain, and OHRQoL (oral health impact profile [OHIP‐14]) data were then collected. The Mann‒Whitney *U* test and Kruskal‒Wallis ANOVA were applied for bivariate analysis. Negative binomial regression was performed to identify significant predictors.

**Results:**

Of the 323 patients who were eligible, 306 participated. The mean age was 39.85 years, and most participants were female (59.5%). OHIP‐14 scores were significantly positively correlated with global general health, oral health, and pain (*p* < 0.001). Sociodemographic variables were not significantly associated with OHRQoL. Individuals with more than one tooth requiring endodontic treatment, multiple decayed teeth, and missing teeth had significantly higher OHIP‐14 scores. Negative binomial regression revealed that the number of teeth requiring endodontic treatment, decayed teeth, and pain were significant predictors of poor OHRQoL.

**Conclusion:**

Patients requiring endodontic treatment experience substantial impairment in OHRQoL, which is influenced primarily by pain and the cumulative oral disease burden rather than by sociodemographic factors.

## 1. Introduction

Almost 3 billion individuals are affected by dental caries, one of the most common chronic diseases worldwide, with untreated dentinal caries in permanent teeth being the primary cause [1]. It is a complex, biofilm‐mediated, and diet‐dependent bacterial infection [2]. Early lesions are mostly limited to enamel and can occasionally be halted with remineralization activities, whereas deeper lesions pose a significantly greater risk. Once dentinal tubules are exposed, bacterial metabolites elicit innate immune responses within the pulp, causing pulpitis. It is characterized by sudden, lingering, and sharp pain and may lead to emergency visits. The clinical symptoms may also include fever, lymphadenopathy, sensitivity, and difficulty chewing, which can lead to emotional and psychological stress in an individual. If untreated, this condition can lead to pulp necrosis and periapical pathologies, including apical periodontitis, abscess formation, and chronic granulomatous inflammation [3].

The treatment modalities include extraction or endodontic treatment. Endodontic treatment aims to eliminate microbial infections from the root canal system while maintaining the structure of the tooth. It is the gold standard for managing irreversible pulpitis and pulpal necrosis [4]. The choice depends on the diagnosis, restorability, patient preference, and cost considerations.

Endodontic pain in a patient is often multifactorial. The perception of pain can be influenced not only by the presence of physical pain but also by psychological, cultural, and socioeconomic factors [5]. The influence of oral disorders on “oral health‐related quality of life” (OHRQoL) is indicated by the fact that patients most frequently experience disturbances in their everyday activities, such as sleeping, eating, and talking [6]. It is a multidimensional construct that evaluates the subjective impact of oral diseases on emotional well‐being, daily functioning, and social interaction.

The OHRQoL helps extend the definition of health to the orofacial domain. Various OHRQoL instruments are available, including the Geriatric Oral Health Assessment Index, oral impacts on daily performance, and the newly developed oral health quality of life for endodontic patients, which provide alternatives for assessing treatment outcomes [7–9]. The most widely used tool is the Oral Health Impact Profile (OHIP), developed by Slade and Spencer [10]. The OHIP originally consisted of 49 questions spread across seven domains [11]. OHIP‐14 was subsequently developed because of the impractical nature of OHIP‐49 in surveys and clinical usage. It comprises seven conceptual domains and has been validated across diverse populations in different languages.

Liu et al. [12] reported that endodontic patients had worse baseline OHRQoL but experienced greater improvement post‐treatment when compared to those receiving periodontal treatment. Endodontic treatment resolved infections and pain, restored function, and improved OHRQoL [13]. Dugas et al. [5] reported that patients who opted for extraction instead of endodontic treatment often experience regret, especially when functional or esthetic concerns arise. Liu et al. [13] and Wigsten et al. [14] studies have reported significant improvements in OHRQoL after endodontic treatment. Zilinskaite‐Petrauskiene and Haug [15] reported that elderly patients had equal or greater benefits from endodontic treatment than younger individuals.

Despite this robust global evidence, there is a significant lack of literature from developing countries, such as India, on OHRQoL in patients requiring endodontic treatment. Cultural attitudes toward pain significantly shape individuals’ healthcare‐seeking behaviors, which subsequently affect their perceptions of oral health. This phenomenon is particularly critical, where factors such as the fear of dental procedures, financial constraints, and limited accessibility often lead to delayed presentation to dentists. Additionally, public awareness and acceptance of endodontic treatment are low. Furthermore, the psychological burden associated with dental pain in most cases remains undervalued, highlighting the need for a more in‐depth understanding of these dynamics. Given the socioeconomic and cultural diversity of India, it is crucial to generate evidence that captures the real‐life implications of dental caries from the patient’s own perspective. The integration of OHRQoL assessment tools into routine clinical practice could support shared decision‐making and enhance treatment planning. Hence, there is a need for research that extends beyond radiographic and clinical metrics to include measures of OHRQoL and to understand the influence of associated factors. Given this background, this study aimed to evaluate the associations of the OHIP‐14 score with sociodemographic and clinical variables among patients requiring endodontic treatment in the Indian context. The null hypothesis would be that there is no association between OHIP‐14 scores and sociodemographic, self‐reported pain, or clinical variables.

## 2. Methodology

We conducted a cross‐sectional study among patients requiring endodontic treatment at the Urban Outreach Comprehensive Dental Clinic in the Udupi District. The Kasturba Medical College and Kasturba Hospital Institutional Ethics Committee approved the project (IEC2 : 262/2024). Prior informed consent was sought from all the participants. The study was conducted from June 2024 to October 2025.

The sample size was estimated on the basis of 12 independent variables, with a target of 25 participants per variable, resulting in an estimated sample size of 300 [16]. These variables included age, sex, years of education, income, number of teeth required for endodontic treatment, pain, decayed, missing, filled tooth index, tooth type, periodontal status, need for post and core, abutment, and periapical index (PAI). Patients who were willing to participate, who required endodontic therapy, and who were aged 18 years or older were included. Participants with acute lesions requiring immediate surgical intervention, those requiring endodontic retreatment, and those who had communication difficulties were excluded. Eligible patients were enrolled using consecutive sampling. Given the outpatient clinical setting and the absence of a defined sampling frame, consecutive sampling was employed to systematically recruit all eligible patients presenting during the study period. This approach minimizes selection bias compared to convenience sampling and provides a reasonable representation of the patient population attending the clinic. A Kannada self‐administered questionnaire was used to gather information on sociodemographics (age, sex, income, and education), OHRQoL, global self‐reported health, oral health, and self‐reported pain before the commencement of endodontic treatment. OHRQoL was evaluated via a validated Kannada version of the OHIP‐14 questionnaire [17]. It was selected due to its established validity and reliability across diverse populations, including the Indian context, and its ability to capture multiple dimensions of OHRQoL, such as pain, psychological discomfort, and social disability. Although condition‐specific instruments like OHRQoL scale for patients undergoing endodontic treatment (OHQE) exist, their applicability is limited by narrower clinical scope (irreversible pulpitis) and lack of cross‐cultural validity. The Kannada language was used as it is a major Dravidian language predominantly spoken in the state of Karnataka, India. The OHIP‐14 consisted of 14 items in seven domains (“functional limitation,” “physical pain,” “psychological discomfort,” “physical disability,” “psychological disability,” “social disability,” and “handicap”) [10]. Each domain had two items rated on a 5‐point Likert scale: “never (0),” “hardly ever (1),” “occasionally (2),” “fairly often (3),” and “very often (4).” The overall score ranged from 0 to 56, with higher scores indicating poorer OHRQoL.

Two global self‐rated questions were used to establish the validity of the OHIP‐14 questionnaire. They include “In general, would you say that your health is…?” and “Overall, how would you rate the health of your teeth and gums?” These were rated on a 5‐point Likert scale: “excellent (1),” “very good (2),” “good (3),” “fair (4),” and “poor (5).” Higher scores indicated poorer self‐rated general and oral health [18].

A self‐reported visual analog scale (VAS) was used to measure pain. It had a 100 mm horizontal line marked at each end, with descriptors representing “no pain” at 0 and “worst imaginable pain” at 100. The participants marked a point on the line that best represented the intensity of their pain [19].

Training and calibration were performed under the supervision of the faculty (Kalyana‐Chakravarthy Pentapati, Shashi Rashmi Acharya, and Ravikiran Ongole), and a single trained examiner (Jishnu Pradeep) performed all the clinical examinations. The decayed, missing, and filled teeth (DMFT) index, a mouth mirror, and a community periodontal index (CPI) probe were used to record dental caries [18]. Loss of attachment (LOA) and the CPI were used to evaluate periodontal health for the tooth that required endodontic treatment [18].

The periapical status of the teeth was evaluated radiographically via the PAI [20]. Intraoral periapical radiographs were taken as part of the routine care provided. The PAI scores were assigned on a scale from 1 to 5, where 1 indicated healthy periapical tissue and 5 denoted severe apical periodontitis with exacerbating features. The prosthetic status of the tooth indicated for endodontic treatment was recorded by observing whether the tooth was indicated for post‐ and core treatments or as an abutment for a future prosthesis. The number of teeth that required endodontic treatment, along with the tooth type (incisors, canines, premolars, and molars), was recorded.

### 2.1. Statistical Analysis

The Statistical Package for the Social Sciences (IBM Corp. Released 2017. IBM SPSS Statistics for Windows, Version 25.0. Armonk, NY, USA) was used to perform the statistical analysis. The *p*‐value of ≤0.05 was considered statistically significant. Normality was assessed using the Kolmogorov–Smirnov test, and the data were found to be non‐normally distributed. Continuous variables, such as age, monthly income, education, decayed teeth, and missing teeth, were categorized via a median split. The mean OHIP scores were compared with sociodemographic variables (age, education, sex, and income) and clinical variables (tooth type, number of teeth requiring endodontic treatment, decayed teeth, missing teeth, CPI, LOA, and teeth indicated for post‐ and core and as abutments for prostheses) via the Mann‒Whitney *U* test/Kruskal‒Wallis ANOVA. Spearman’s correlation coefficient was used to assess the correlation between the OHIP questionnaire and global self‐rated questions, as was the VAS scale for pain. The variances of the independent variables were less than those of the means. Hence, negative binomial regression analysis was performed to identify significant predictors of poor OHRQoL. Cronbach’s alpha was used to evaluate the internal consistency of the OHIP‐14 questionnaire.

## 3. Results

**Figure 1:**
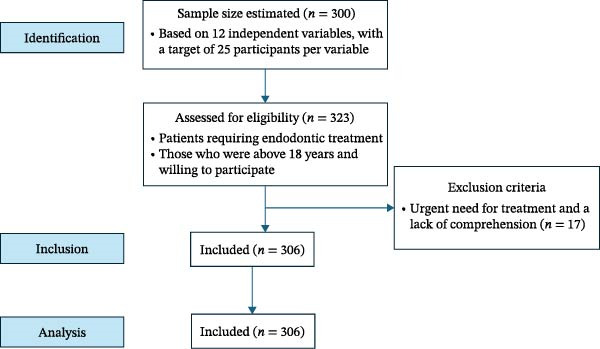
Flow diagram of participant selection.

A total of 323 patients were approached to participate in the study, and 306 responded. Twenty‐three patients were excluded because of an urgent need for treatment and a lack of comprehension (Figure 1). The mean age was 39.85 years. Most participants were female (*n* = 182; 59.5%). The median monthly income was USD 120. The mean numbers of decayed teeth, missing teeth, and teeth requiring endodontic treatment were 2.37, 1.28, and 1.18, respectively (Table 1).

**Table 1:** Distribution of sociodemographic variables.

Variable	Mean	Median	SD
Age	39.85	39	16.25
Monthly income INR (USD)	16,404 (197)	10,000 (120)	22,757 (273)
Decayed teeth	2.37	2	1.51
Missing teeth	1.28	0	2.07
Filled teeth	2.06	2	2.01
Number of teeth indicated for endodontic treatment	1.18	1	0.54
Total mean OHIP score	10.59	8.00	8.53
Pain	33.33	30.00	25.61

Abbreviations: INR, Indian National Rupee; OHIP, Oral Health Impact Profile; SD, standard deviation; USD, United States Dollar.

The majority of patients reported occasional pain (44.8%), had to interrupt their meals (23.9%) or had difficulty relaxing (23.9%), and had difficulty eating any type of food (29.1%) (Table 2).

**Table 2:** Distribution of responses of OHIP questionnaire.

Questionnaire item	Never		Hardly ever		Occasionally		Fairly often		Very often	
	*N*	(%)	*N*	(%)	*N*	(%)	*N*	(%)	*N*	(%)
“Have you had trouble pronouncing any words because of problems with your teeth or mouth?”	226	73.90	47	15.40	29	9.50	2	0.70	2	0.70
“Have you felt that your sense of taste has worsened because of problems with your teeth or mouth?”	217	70.90	49	16.00	37	12.10	2	0.70	1	0.30
“Have you had painful aching in your mouth?”	48	15.70	61	19.90	137	44.80	43	14.10	17	5.60
“Have you found it uncomfortable to eat any foods because of problems with your teeth or mouth?”	119	38.90	64	20.90	89	29.10	27	8.80	7	2.30
“Have you been self‐conscious because of your teeth or mouth?”	176	57.50	69	22.50	41	13.40	16	5.20	4	1.30
“Have you felt tense because of problems with your teeth or mouth?”	151	49.30	71	23.20	63	20.60	13	4.20	8	2.60
“Has your diet been unsatisfactory because of problems with your teeth or mouth?”	167	54.60	64	20.90	62	20.30	10	3.30	3	1.00
“Have you had to interrupt meals because of problems with your teeth or mouth?”	169	55.20	45	14.70	73	23.90	16	5.20	3	1.00
“Have you found it difficult to relax because of problems with your teeth or mouth?”	141	46.10	66	21.60	73	23.90	18	5.90	8	2.60
“Have you been a bit embarrassed because of problems with your teeth or mouth?”	177	57.80	57	18.60	59	19.30	6	2.00	7	2.30
“Have you been a bit irritable with other people because of problems with your teeth or mouth?”	205	67.00	60	19.60	34	11.10	3	1.00	4	1.30
“Have you had difficulty doing your usual jobs because of problems with your teeth or mouth?”	190	62.10	49	16.00	52	17.00	11	3.60	4	1.30
“Have you felt that life in general was less satisfying because of problems with your teeth or mouth?”	232	75.80	39	12.70	29	9.50	6	2.00	0	0.00
“Have you been totally unable to function because of problems with your teeth or mouth?”	216	70.60	41	13.40	40	13.10	5	1.60	4	1.30

The concurrent (criterion) validity of the OHIP questionnaire was evaluated against the global single‐item questionnaire for general health and oral health. There was a significant, moderate positive correlation between total OHIP scores and global general and oral health ratings (*r* = 0.45, *p* < 0.001, and *r* = 0.46, *p* < 0.001, respectively). The convergent validity of the OHIP questionnaire was evaluated against self‐reported pain via the VAS. There was a significant positive correlation between total OHIP score and self‐reported pain (*r* = 0.25; *p* < 0.001) (Table 3). Cronbach’s alpha for internal consistency reliability was 0.89, which was above the acceptable level.

**Table 3:** Concurrent (criterion) validity of the OHIP questionnaire with global single‐item questionnaire for general health, oral health, and pain scores using visual analog scale.

Parameter	General health	Oral health	VAS pain
Spearman’s rho correlation coefficient	0.45 ^∗∗^	0.46 ^∗∗^	0.25 ^∗∗^
*p*‐Value	<0.001	<0.001	<0.001

Abbreviations: OHIP, Oral Health Impact Profile; VAS, visual analog scale.

^∗∗^Denotes statistical significance at *p* < 0.01.

There were no significant differences in the mean total OHIP scores with respect to age, sex, education, or monthly income (Table 4). Also, there were no significant differences in the mean total OHIP scores with respect to tooth type, periodontal variables (CPI and LOA), or the need for post‐core or abutment or PAI. However, individuals requiring endodontic treatment for more than one tooth, those with multiple decayed teeth, or those with missing teeth reported significantly higher total OHIP scores than their counterparts. (Table 5).

**Table 4:** Comparison of total OHIP scores with demographic variables.

Variable	Total OHIP			*p*‐Value
	Mean	SD	*N*	
Age in years				
Upto 39	10.28	8.42	155	0.535
39 and more	10.91	8.65	151	
Gender				
Male	11.30	9.46	124	0.617
Female	10.1	7.82	182	
Education in years				
Upto 12	9.83	8.22	166	0.096
>12	11.49	8.82	140	
Monthly income (INR)				
Upto 120 USD (10,000 INR)	9.92	7.81	146	0.463
>120 USD (>10,000 INR)	11.19	9.12	160	

Abbreviations: INR, Indian National Rupee; OHIP, Oral Health Impact Profile; SD, standard deviation; USD, United States Dollar.

**Table 5:** Comparison of total OHIP scores with clinical variables.

Variable		Total OHIP			*p*‐Value
		Mean	SD	*N*	
Tooth type	Anteriors	11.08	7.66	37	0.464
	Posteriors	10.52	8.65	269	
Number of teeth for endodontic treatment	1	10.01	8.15	264	0.006 ^∗^
	>1	14.24	9.97	42	
Decayed teeth	0–2	9.45	7.65	200	0.006 ^∗^
	>2	12.74	9.65	106	
Missing teeth	Nil	9.51	7.95	154	0.029 ^∗^
	Present	11.68	8.97	152	
CPI	0	9.77	8.13	173	0.08
	1	11.24	8.68	122	
	2	16.27	10.85	11	
LOA	0	10.51	8.48	287	0.66
	1	11.74	9.38	19	
Post and core	Yes	11.09	7.74	11	0.67
	No	10.57	8.57	295	
Abutment	Yes	11.92	9.20	25	0.51
	No	10.47	8.47	281	
PAI score	1	10.33	8.75	249	0.154
	2	11.00	7.47	38	
	3	14.07	7.55	14	
	4	10.60	7.13	5	

Abbreviations: CPI, community periodontal index; LOA, loss of attachment; OHIP, Oral Health Impact Profile; PAI, periapical index; SD, standard deviation.

“ ^∗^” denotes statistical significance.

Negative binomial regression analysis was performed to identify the significant predictors. (Table 4). The number of teeth requiring endodontic treatment (rate ratio [RR]: 1.4), decayed teeth (RR: 1.35), and pain (RR: 1.01) were significant predictors of poor OHRQoL (Table 6).

**Table 6:** Association of total OHIP scores with clinical variables.

Predictor	RR (95% CI)	*p*‐Value
Number of teeth for endodontic treatment	1.4 (1.02−1.99)	0.04 ^∗^
Decayed teeth	1.35 (1.05−1.72)	0.017 ^∗^
Missing teeth	1.23 (0.97−1.55)	0.085
Pain	1.01 (1–1.01)	0.002 ^∗^

Abbreviations: CI, confidence interval; OHIP, Oral Health Impact Profile; RR, rate ratio.

“ ^∗^” denotes statistical significance.

## 4. Discussion

Recently, there has been a shift in focus from evaluating diseases and conditions on the basis of patients’ perceptions rather than their clinical presentations [5, 21]. Only a few studies have been conducted globally that have evaluated OHRQoL and various predictors among patients who require endodontic treatment, with no studies in the Indian context [13, 14, 22]. Hence, we conducted this study to evaluate the OHRQoL and the associated socioeconomic and clinical predictors among patients requiring endodontic treatment.

The median age was 39 years, which was lower than that reported in previous studies [13, 14]. These findings suggest that younger participants are more likely to seek treatment than the elderly participants in this population. This study also revealed that most participants seeking treatment were females, a finding consistent with previous studies. None of the sociodemographic variables (age, sex, monthly income, or education) was significantly associated with OHRQoL, as observed in earlier studies [5, 13, 22, 23]. The impact of oral conditions on OHRQoL appears to be due to immediate clinical symptoms rather than sociodemographic factors, suggesting that patients tend to seek care at symptomatic stages irrespective of their socioeconomic background. However, Zilinskaite‐Petrauskiene and Haug [15], Guimarães et al. [24], and Khoo et al. [25] have reported that females had poorer OHRQoL than males. Zilinskaite‐Petrauskiene and Haug [15] have reported that younger people had poorer OHRQoL than the elderly. These differences may reflect variations in health awareness, pain perception, and healthcare utilization across populations. In the Indian scenario, delayed care‐seeking and a tendency to prioritize symptomatic relief could have attenuated the influence of sociodemographic variables on OHRQoL. Clinical variables, such as periodontal status, were not significantly associated with OHRQoL, contrary to the expectations [13, 22]. This may be attributed to the relatively younger study population, in whom periodontal disease is typically less severe. Previous studies have shown that the type of tooth has no significant association with OHRQoL; a similar trend was observed in our study [13, 22–26]. This suggests that patients may experience impaired OHRQoL irrespective of the type of tooth requiring endodontic treatment. However, Zilinskaite‐Petrauskiene and Haug [15] reported poorer OHRQoL among patients undergoing endodontic treatment in anterior teeth and premolars compared to molars, which may reflect population‐specific differences in esthetic concerns and functional expectations.

Many sociodemographic and clinical variables showed no significant differences with the total OHIP scores. This highlights that the need for endodontic treatment has a comparable impact across various levels of these variables, suggesting that OHRQoL is driven more by immediate clinical burden than by sociodemographic variables. Notably, the need for endodontic treatment in multiple teeth and multiple decayed teeth was shown to have a significant association with OHRQoL, indicating a direct and cumulative impact of disease severity on the OHRQoL. These findings highlight that increasing oral disease burden amplifies functional limitations and discomfort, thereby worsening OHRQoL.

The mean pain score was 33.3, which was slightly higher than that in the previous study. As in a previous study, the pain reported was of less intensity [13, 22]. This may be explained by factors such as prior analgesic use or delays in seeking care, leading to partial subsidence of acute symptoms. Nevertheless, pain remains a key driver of treatment‐seeking behavior and has a substantial impact on daily functioning, thereby contributing significantly to impaired OHRQoL. The mean number of decayed teeth was greater than that reported in previous studies. It is significantly associated with poor OHRQoL, contrary to earlier findings [13, 22]. The significant association between decayed teeth and poor OHRQoL highlights the role of untreated caries as a source of pain and functional limitation, resulting in poor OHRQoL.

Research has demonstrated that endodontic problems have a significant effect on OHRQoL [13, 22, 26]. Our study identified three significant clinical predictors, namely, the number of teeth requiring endodontic treatment, pain, and the number of decayed teeth. Similarly, Liu et al. [13] reported a significant association between multiple endodontic treatments and pain with OHRQoL. These findings imply that multiple oral problems and pain could have a cumulative impact on the OHRQoL. Liu et al. [13] reported that age was significantly associated with the OHRQoL. This may be due to differences in population structure, particularly the inclusion of older individuals [13]. Zilinskaite‐Petrauskiene and Haug [15] have reported that patients with pain, younger age, female sex, and the need for endodontic treatment in anterior or premolar teeth had poor OHRQoL, indicating that both clinical severity and population characteristics can influence outcomes. The number of teeth requiring endodontic treatment can be an indicator of symptomatic burden that can impair the OHRQoL. The observed associations help us to understand individual perception of pain, and disease severity can have potential influence on OHRQoL. Collectively, these factors highlight the need for early diagnosis and prompt treatment to prevent the deterioration of the OHRQoL.

The use of a validated questionnaire in the local language guaranteed that the domains under research were measured with established validity and reliability. While condition‐specific instruments like the OHQE may offer greater sensitivity, the use of OHIP‐14 enabled broader assessment across a range of endodontic conditions, enhancing the generalizability of the findings. Integrating objective clinical evaluations with patient‐reported outcomes enhanced the comprehensiveness of the evaluation and allowed for a more nuanced view that considers both clinical status and perceived OHRQoL. The inclusion of patient‐reported outcome measures adds significant value to the study since they reflect the subjective components of health and functioning that are not adequately captured by clinical indicators alone. A notable drawback is the use of a self‐report questionnaire, which is naturally subject to human perception and may introduce various biases, such as social desirability, exaggerated reactions, or central tendencies. We could not include patients undergoing urgent endodontic treatment as they may have a greater impairment in OHRQoL. Only 13.7% of the participants required endodontic treatment in more than one teeth which limited the feasibility of stratified analysis in this subgroup. The inclusion of diverse diagnostic categories could have introduced some degree of variability in self‐reported pain scores. The strength of this study lies in its large sample size, which encompasses a range of clinical and sociodemographic factors, providing sufficient statistical power to address the research objectives.

The results highlight the need for the inclusion of OHRQoL assessment into routine dental care and public health screening to help policymakers and clinicians understand the impact of oral diseases. It also emphasizes the need for strengthening prevention‐based strategies and prompt conservation of teeth to reduce the disease progression and decrease the ORHQoL burden. Such patient‐reported outcomes can guide resource allocation toward preventive interventions that address both disease and its implications. Future recommendations include the integration of OHRQoL measures into treatment planning and routine dental care, training clinicians in the use and interpretation of OHRQoL tools, and promoting prospective research to strengthen patient‐centered oral health care. Future studies should focus on prospective study designs evaluating pre‐ and post‐treatment OHRQoL with condition‐specific questionnaires. Such studies would yield representative data, which can inform academic research, guide clinical practice, and support policymakers in improving patient‐centered outcomes.

## Author Contributions

Conceptualization: Jishnu Pradeep, Kalyana‐Chakravarthy Pentapati, and Shashi Rashmi Acharya. Data curation: Jishnu Pradeep and Kalyana‐Chakravarthy Pentapati. Formal analysis: Jishnu Pradeep and Kalyana‐Chakravarthy Pentapati. Validation: Jishnu Pradeep, Shashi Rashmi Acharya, Ravikiran Ongole. Methodology: Jishnu Pradeep, Shashi Rashmi Acharya, Kalyana‐Chakravarthy Pentapati, and Ravikiran Ongole. Writing – original draft preparation: Jishnu Pradeep, Kalyana‐Chakravarthy Pentapati, and Shashi Rashmi Acharya. Writing – review and editing: Jishnu Pradeep, Kalyana‐Chakravarthy Pentapati, Shashi Rashmi Acharya, and Ravikiran Ongole.

## Funding

This study did not receive any specific funding.

## Disclosure

All authors have read and approved the final version of the manuscript. Corresponding author had full access to all of the data in this study and takes complete responsibility for the integrity of the data and the accuracy of the data analysis.

## Ethics Statement

Kasturba Medical College and Kasturba Hospital Institutional Ethics Committee approved the project. Prior informed consent was sought from all the participants.

## Conflicts of Interest

The authors declare no conflicts of interest.

## Data Availability

The data that support the findings of this study are available from the corresponding author upon reasonable request.

## References

[1] World Health Organization , Global Oral Health Status Report: Towards Universal Health Coverage for Oral Health by 2030: Summary of the WHO European Region, Dental Abstracts. (2022) 57, 1–120.

[2] Pitts N. B. , Zero D. T. , and Marsh P. D. , et al.Dental Caries, Nature Reviews Disease Primers. (2017) 3, no. 1, 10.1038/nrdp.2017.30, 17030. 28540937

[3] Seltzer S. , Bender I. B. , and Ziontz M. , The Dynamics of Pulp Inflammation: Correlations Between Diagnostic Data and Actual Histologic Findings in the Pulp, Oral Surgery, Oral Medicine, Oral Pathology. (1963) 16, no. 8, 969–977, 10.1016/0030-4220(63)90201-9. 14049126

[4] Gutmann J. L. , Clinical, Radiographic, and Histologic Perspectives on Success and Failure in Endodontics, Dental Clinics of North America. (1992) 36, no. 2, 379–392, 10.1016/S0011-8532(22)02502-2. 1572505

[5] Dugas N. N. , Lawrence H. P. , Teplitsky P. , and Friedman S. , Quality of Life and Satisfaction Outcomes of Endodontic Treatment, Journal of Endodontics. (2002) 28, no. 12, 819–827, 10.1097/00004770-200212000-00007. 12489651

[6] Locker D. and Allen F. , What Do Measures of “Oral Health-Related Quality of Life” Measure?, Community Dentistry and Oral Epidemiology. (2007) 35, no. 6, 401–411, 10.1111/j.1600-0528.2007.00418.x. 18039281

[7] Atchison K. A. and Dolan T. A. , Development of the Geriatric Oral Health Assessment Index, Journal of Dental Education. (1990) 54, no. 11, 680–687, 10.1002/j.0022-0337.1990.54.11.tb02481.x. 2229624

[8] Åstrøm A. N. and Okullo I. , Validity and Reliability of the Oral Impacts on Daily Performance (OIDP) Frequency Scale: A Cross-Sectional Study of Adolescents in Uganda, BMC Oral Health. (2003) 3, no. 1, 10.1186/1472-6831-3-5, 5. 12943555 PMC212323

[9] Arifin F. A. , Matsuda Y. , and Kanno T. , Development and Validation of Oral Health-Related Quality of Life Scale for Patients Undergoing Endodontic Treatment (OHQE) for Irreversible Pulpitis, Healthcare. (2023) 11, no. 21, 10.3390/healthcare11212859, 2859. 37958003 PMC10648889

[10] Slade G. D. , Derivation and Validation of a Short-Form Oral Health Impact Profile, Community Dentistry and Oral Epidemiology. (1997) 25, no. 4, 284–290, 10.1111/j.1600-0528.1997.tb00941.x. 9332805

[11] Slade G. D. and Spencer A. J. , Development and Evaluation of the Oral Health Impact Profile, Community Dental Health. (1994) 11, no. 1, 3–11. 8193981

[12] Liu P. , McGrath C. , and Cheung G. S. P. , Quality of Life and Psychological Well-Being Among Endodontic Patients: A Case-Control Study, Australian Dental Journal. (2012) 57, no. 4, 493–497, 10.1111/j.1834-7819.2012.01722.x. 23186576

[13] Liu P. , McGrath C. , and Cheung G. , What Are the Key Endodontic Factors Associated With Oral Health-Related Quality of Life?, International Endodontic Journal. (2014) 47, no. 3, 238–245, 10.1111/iej.12139. 23800195

[14] Wigsten E. , Kvist T. , and Jonasson P. , et al.Comparing Quality of Life of Patients Undergoing Root Canal Treatment or Tooth Extraction, Journal of Endodontics. (2020) 46, no. 1, 19–28, 10.1016/j.joen.2019.10.012. 31843125

[15] Zilinskaite-Petrauskiene I. and Haug S. R. , A Comparison of Endodontic Treatment Factors, Operator Difficulties, and Perceived Oral Health–Related Quality of Life Between Elderly and Young Patients, Journal of Endodontics. (2021) 47, no. 12, 1844–1853, 10.1016/j.joen.2021.08.017. 34499888

[16] Bland J. M. and Altman D. G. , Correlation, Regression, and Repeated Data, BMJ. (1994) 308, no. 6933, 10.1136/bmj.308.6933.896, 896. 8173371 PMC2539813

[17] Acharya S. , Bhat P. V. , and Acharya S. , Factors Affecting Oral Health-Related Quality of Life Among Pregnant Women, International Journal of Dental Hygiene. (2009) 7, no. 2, 102–107, 10.1111/j.1601-5037.2008.00351.x. 19416092

[18] World Health Organization , Oral Health Surveys Basic Methods, 2013, 5th edition, WHO.

[19] Huskisson E. C. , Measurement of Pain, The Lancet. (1974) 304, no. 7889, 1127–1131, 10.1016/S0140-6736(74)90884-8. 4139420

[20] Ørstavik D. , Kerekes K. , and Eriksen H. M. , The Periapical Index: A Scoring System for Radiographic Assessment of Apical Periodontitis, Dental Traumatology. (1986) 2, no. 1, 20–34, 10.1111/j.1600-9657.1986.tb00119.x. 3457698

[21] Wright W. G. , Jones J. A. , Spiro A. , Rich S. E. , and Kressin N. R. , Use of Patient Self-Report Oral Health Outcome Measures in Assessment of Dental Treatment Outcomes, Journal of Public Health Dentistry. (2009) 69, no. 2, 95–103, 10.1111/j.1752-7325.2008.00106.x. 19054312 PMC3539755

[22] Liu P. , McGrath C. , and Cheung G. S. P. , Improvement in Oral Health-Related Quality of Life after Endodontic Treatment: A Prospective Longitudinal Study, Journal of Endodontics. (2014) 40, no. 6, 805–810, 10.1016/j.joen.2014.02.008. 24862707

[23] Jaiswal A. , Zaveri M. , Unjia A. , Shah S. , Langaliya A. , and Kumar S. , Patient Satisfaction and Oral Health-Related Quality of Life Before and After Endodontic Treatment: A Longitudinal Study, Journal of Pharmacy and Bioallied Sciences. (2023) 15, no. Suppl 2, S1000–S1002, 10.4103/jpbs.jpbs_253_23. 37694006 PMC10485523

[24] Guimarães L. S. , da Silva E. A. B. , Hespanhol F. G. , Tavares MLCDA. , Antunes L. A. A. , and Antunes L. S. , Impact of Root Canal Treatment on Oral Health-Related Quality of Life: A Prospective Cohort Study, Brazilian Oral Research. (2025) 39, 10.1590/1807-3107bor-2025.vol39.126, 126. PMC12683721 41379131

[25] Khoo S. T. , Ode W. , Lopez V. , Yu V. S. H. , Lai C. , and Lui J. N. , Factors Influencing Quality of Life After Surgical and Nonsurgical Interventions of Persistent Endodontic Disease, Journal of Endodontics. (2020) 46, no. 12, 1832–1840, 10.1016/j.joen.2020.08.020. 32898556

[26] Hamasha A. A. and Hatiwsh A. , Quality of Life and Satisfaction of Patients After Nonsurgical Primary Root Canal Treatment Provided by Undergraduate Students, Graduate Students and Endodontic Specialists, International Endodontic Journal. (2013) 46, no. 12, 1131–1139, 10.1111/iej.12106. 23560436

